# Regulation of CFTR Biogenesis by the Proteostatic Network and Pharmacological Modulators

**DOI:** 10.3390/ijms21020452

**Published:** 2020-01-10

**Authors:** Samuel Estabrooks, Jeffrey L. Brodsky

**Affiliations:** Department of Biological Sciences, University of Pittsburgh, Pittsburgh, PA 15260, USA; ske12@pitt.edu

**Keywords:** cystic fibrosis, molecular chaperone, proteasome, ubiquitin, protein folding, protein trafficking, degradation, ERAD

## Abstract

Cystic fibrosis (CF) is the most common lethal inherited disease among Caucasians in North America and a significant portion of Europe. The disease arises from one of many mutations in the gene encoding the cystic fibrosis transmembrane conductance regulator, or CFTR. The most common disease-associated allele, F508del, along with several other mutations affect the folding, transport, and stability of CFTR as it transits from the endoplasmic reticulum (ER) to the plasma membrane, where it functions primarily as a chloride channel. Early data demonstrated that F508del CFTR is selected for ER associated degradation (ERAD), a pathway in which misfolded proteins are recognized by ER-associated molecular chaperones, ubiquitinated, and delivered to the proteasome for degradation. Later studies showed that F508del CFTR that is rescued from ERAD and folds can alternatively be selected for enhanced endocytosis and lysosomal degradation. A number of other disease-causing mutations in CFTR also undergo these events. Fortunately, pharmacological modulators of CFTR biogenesis can repair CFTR, permitting its folding, escape from ERAD, and function at the cell surface. In this article, we review the many cellular checkpoints that monitor CFTR biogenesis, discuss the emergence of effective treatments for CF, and highlight future areas of research on the proteostatic control of CFTR.

## 1. Cystic Fibrosis

### 1.1. Pathology

Originally identified as a distinct pathology over eighty years ago, CF was initially defined as a lethal, primarily digestive disorder affecting newborns [1]. Described at the time as “cystic fibrosis of the pancreas”, postmortem observations of infants succumbing to the disease revealed thick, immobile mucus lining the digestive tract, which blocked entry of pancreatic enzymes into the intestine via the pancreatic duct. As a result of severe pancreatic insufficiency, infants with CF tended to exhibit meconium ileus (intestinal obstruction), perforated intestines, abnormal stools, and a failure to thrive, rarely surviving for more than a few months. Fortunately, CF patients today have ready access to pancreatic enzyme replacement therapy (PERT), which along with multivitamins alleviates digestive symptoms and restores typical nutrient absorption [2]. With rigorous newborn genetic screening, many CF patients are now diagnosed and prescribed PERT before gastrointestinal symptoms even have the opportunity to develop.

Further clinical characterization led to the observation that CF patients also acquire and die from respiratory infections such as pneumonia and bronchitis, provided that these individuals did not first succumb to intestinal blockage [3]. As care improved and patients gradually lived beyond infancy into childhood, it became clear that pulmonary dysfunction is a hallmark of CF. Just as within the digestive tract, CF causes the layer of mucus coating tissues of the respiratory tract to become dehydrated, immobile, and increasingly acidic [4,5]. Because mucus fluidity is critical for maintaining the efflux of inhaled microorganisms from the respiratory tract, the lungs of CF patients gradually become infected with pathogenic bacteria and fungi [6]. Once these intractable infections are established, they permanently activate an immune response [7], causing persistent inflammation of respiratory tissue that drastically reduces lung function, which is typically measured by forced expiratory volume (FEV) [8]. Although the makeup of lung flora varies widely by patient, *Pseudomonas aeruginosa*, *Staphylococcus aureus*, and non-Tuberculosis mycobacterial species are among the most common bacterial pathogens that colonize CF lungs [9,10]. Intriguingly however, it is worth noting that even without bacterial colonization, CF ferrets (an animal model that successfully recapitulates CF respiratory symptoms) still exhibit inflammatory lung disease [11], suggesting that bacterial colonization—while associated with poor prognosis—may not be required to activate an immune response in CF. Regardless, in an effort to dislodge mucus plugs, ease inflammation, and reduce pathogenic burden, CF patients are frequently prescribed mucolytics, corticosteroids, inhaled hypertonic saline, as well as oral or inhaled antibiotics [12]. Additionally, numerous techniques have been designed to physically clear airways, such as forced expiration, exercise, and the use of a high frequency chest wall oscillation (HFCWO) vest [13,14]. Even so, the vast majority of CF patients ultimately endure pulmonary exacerbations, periods of intense pathogenic burden and sharply reduced pulmonary function requiring hospitalization. Though pulmonary exacerbations are typically the terminal event for CF patients, therapeutic advancements made over the past several decades have steadily slowed the rate of respiratory decline in patients and reduced the frequency of exacerbations, enabling patients to lead longer, healthier lives (see Section 3 below). Indeed, only recently did the median age of CF patients surpass 18 years of age for the first time [15], shifting how this once pediatric disease is now viewed and treated.

As patients live longer, however, additional complications not originally appreciated as symptoms of CF have begun to emerge. For example, even with prolonged replacement of pancreatic function, ~40% of CF patients are diagnosed with gastroesophageal reflux disease (GERD), for which many individuals additionally take acid blockers or proton pump inhibitors [16]. Furthermore, almost one-third of adult CF patients exhibit glucose intolerance and have been further diagnosed with CF-related diabetes (CFRD), which requires regular insulin injections [17,18]. Although less frequent, some CF patients also display unusual liver function, generally referred to as CF liver disease [19]. Moreover, as male fertility also relies on mucus-lined ducts, nearly all male patients have congenital bilateral absence of the vas deferens (CBAVD) and are rendered infertile, a topic of concern as a growing number CF patients survive into adulthood [20,21]. Finally, approximately one-quarter of all CF adults report suffering from depression or anxiety [22,23], indicating that this disease not only takes a physical toll upon those afflicted, but a psychological one as well. Overall, how CF affects the health of most tissues throughout the body remains incompletely understood but is of significant concern as the CF population continues to age.

### 1.2. CFTR Structure and Function

Early studies into the epidemiology of CF noted that the disease affects males and females at equal rates, but appeared to be hereditary as it tracked within certain families at a much higher frequency than the general population [3]. Decades later, the genetic determinant would be pinpointed to a single locus on chromosome 7, a ~6500 base pair gene thereafter known as the cystic fibrosis transmembrane conductance regulator (CFTR) [24,25].

**Figure 1:**
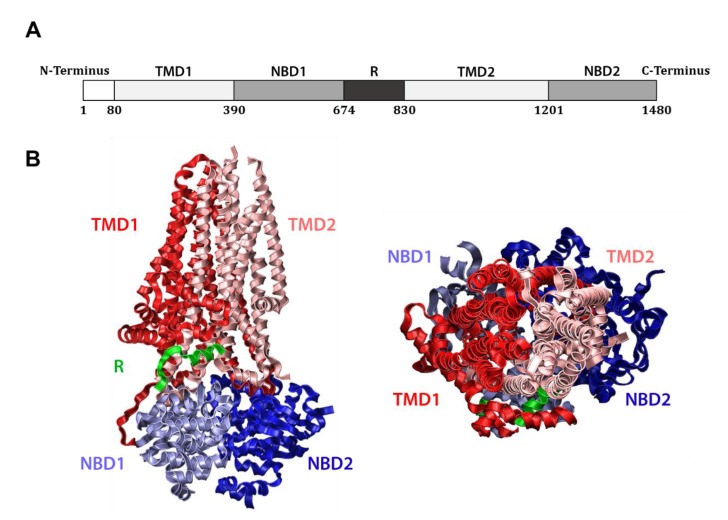
Structure of CFTR: (**A**) Linear schematic of CFTR domain organization; (**B**) Side view (left) and top view (right) depicting the Cryo-EM structure of phosphorylated, ATP-bound CFTR (PDB ID: 6MSM) [32]. Note that only part of the R domain is included, as the inherent flexibility of this domain limits its visibility by Cryo-EM.

The CFTR gene encodes a 1480 amino acid, ~168 kDa protein which is classified as a member of the ATP-binding cassette (ABC) transporter superfamily, a set of interrelated transmembrane proteins found in both eukaryotes and prokaryotes that bind ATP and promote substrate transport across cellular membranes. As such, CFTR (alternately known as ABC subfamily C member 7) bears close structural homology to other ABC transporters, including certain bacterial multidrug resistance pumps [26]. Like these, CFTR contains two hydrophobic transmembrane domains (TMDs) that anchor the protein in the membrane, as well as two nucleotide binding domains (NBDs) that reside in the cytosol (Figure 1A). Unlike other ABC transporters, CFTR also harbors a regulatory (R) domain between NBD1 and TMD2, which provides an extra layer of control over its activity. Specifically, the R domain must be phosphorylated by Protein Kinase A in a cyclic AMP (cAMP)-dependent manner for CFTR to fully function [27]. Phosphorylation displaces the flexible R domain, favors NBD interaction, and exposes the protein’s positively charged central pore [28,29] (Figure 1B). Intimate association between the NBDs allows for two, shared ATP-binding sites to be established at their interface. Notably, each site is composed of the Walker A (GXXGXGKS/T) and Walker B (ΦΦΦΦD, where Φ is hydrophobic) motifs of one domain and the signature motif (LSGGQ) of the other, which together coordinate the phosphate groups of ATP and bind an associated magnesium ion [30]. Only one of these composite sites is active in CFTR however, as the site established by the Walker motifs of NBD1 and the signature motif of NBD2 contains a nonconserved signature motif (LSHGH), causing this site to bind but fail to catalyze ATP. These shared ATP binding sites are required for ATP hydrolysis, and the resulting conformational change transitions CFTR between closed and open states, effecting the movement of substrates through a central pore [28,31,32,33].

The pore formed by CFTR also differs from other ABC transporters. Uniquely, CFTR facilitates the movement of anions across a cellular membrane, the only ABC transporter known to do so [34]. Moreover, CFTR functions as a channel rather than as a bona fide transporter. Unlike multidrug resistance pumps, which hydrolyze ATP to actively pump potentially toxic compounds out of cells against a chemical gradient [35], CFTR utilizes ATP to transition between the open and closed states but cannot specify the direction of flow. Therefore, when CFTR opens at the surface of epithelial cells, anions flow passively down their electrochemical gradient. In most epithelial tissues, such as those in the lungs and digestive tract, this corresponds to an efflux of chloride and bicarbonate from within cells into the extracellular space. Chloride release generates a motive force for the concomitant transport of water, thus hydrating the apical surface of these organs. By additionally releasing bicarbonate from cells, CFTR also mitigates acidity in the respiratory tract [36]. Therefore, when CF patients inherit dysfunctional variants of CFTR, the mucus lining the airways becomes dehydrated and increasingly acidic, providing an ideal environment to support bacterial growth [37].

In contrast to pulmonary tissue, the extracellular concentration of chloride is typically higher than the intracellular concentration in sweat ducts [38]. Consequently, CFTR opening instead triggers a net influx of chloride from the extracellular environment. As a result, electrolytes tend to linger in significantly higher quantities on the skin of CF patients. Though of little pathological consequence, it was the recognition of this trait that brought about sweat chloride testing as the first bioassay used to distinguish CF from other respiratory ailments [39]. Measurements of sweat chloride continue to be used as a reliable means of assessing the efficacy of therapeutics that target CFTR [40].

### 1.3. Inheritance of Dysfunctional CFTR Variants Causes CF

Mutations in CFTR are thought to have been selected for amongst Bronze Age peoples of Western Europe as an adaptation against secretory diarrheal diseases, such as cholera [41,42]. Notably, individuals carrying one defective allele are less prone to severe dehydration. To date, over 300 distinct CFTR mutations have been identified that cause CF (https://cftr2.org). These mutations have been binned into five separate classes based on their molecular defects [43].

Class I variants are those which produce either an incomplete CFTR protein or no protein at all. This typically occurs due to a mutation in CFTR that causes translation to prematurely terminate, but can also occur due to the insertion or deletion of base pairs that shift the CFTR reading frame. Class I variants, such as G542X and W1282X CFTR, produce truncated channels and account for around 15% of CFTR variants carried by CF patients in the United States [15,44]. Currently, these have proven difficult to “correct” therapeutically (also see Section 3).

Class II variants are full length CFTR channels, but include a mutation which causes domains within the protein to misfold, preventing the channel from trafficking to the cell surface. Class II variants are the most frequently occurring mutations amongst CF patients. Indeed, 86% of American CF patients encode at least one copy of the most prominent Class II variant, F508del CFTR [15]. The deletion of phenylalanine 508 from NBD1 causes this domain to misfold and compromises interactions both between NBD1 and NBD2 and between NBD1 and the transmembrane domains [45,46,47,48,49,50]. Based on its frequency in the population, there has been significant interest in correcting the molecular defects associated with this variant (see Section 3.1). Other more infrequent variants, such as G85E (0.7% of patients) and N1303K (2.4% of patients), also impair CFTR folding but reside within TMD1 and NBD2, respectively.

Both Class III and IV variants instead affect the function of CFTR channels at the cell surface. Class III variants traffic normally to the surface but are gated incorrectly. These variants, which include G551D CFTR (4.5% of patients), cannot open or exhibit reduced open probabilities. As such, Class III variants fail to conduct sufficient chloride and bicarbonate ions across epithelial cell membranes. The activity of some of these mutations is significantly improved by small molecule “potentiator” compounds that are used clinically [51] (see Section 3.2). While Class IV variants closely resemble Class III variants in terms of their mitigated function, they instead conduct ion currents weakly due to mutations that misshape or confer an unfavorable electrochemical charge within the channel’s central pore.

Finally, Class V variants include those that produce functional CFTR channels but in quantities too small to effectively facilitate anion exchange across cell membranes. For instance, A455E CFTR (0.6% of patients) is as functional as wild-type CFTR, but causes a moderate form of CF due to its inherently lower level of protein synthesis [52].

While these classifications exist to better guide therapeutic strategies to address the underlying molecular defect of individual variants, it has become increasingly clear that dysfunctional variants can fall into multiple classes [53]. For instance, F508del CFTR has been characterized as a Class II, Class III, and Class IV variant. In addition to the misfolding defect of this variant (see above), any F508del channels that do manage to fold and reach the cell surface conduct current across the membrane with reduced efficiency [54,55,56]. Thus, optimal treatments for individuals carrying the F508del allele require drug combinations that target each of these distinct defects (see Section 3.3).

## 2. CFTR is Subject to Multiple Protein Quality Control Checkpoints

Like numerous other cellular proteins that traffic from the endoplasmic reticulum (ER) to the cell surface through the secretory pathway, CFTR is perpetually monitored by protein quality control (PQC) factors. Quality control “decisions” regarding CFTR are generally made at two key points in the cell, either (1) at the ER as CFTR is synthesized, and/or (2) in post-ER compartments after CFTR has folded and trafficked from the ER. How different CFTR variants interact with the PQC machinery at each of these locations has major ramifications for the disease severity associated with distinct variants, as well as the approaches used to therapeutically treat the disease. The PQC machinery, the pathways that lead to the degradation or folding of cellular proteins, and the stress responses that are triggered when misfolded proteins accumulate have collectively been referred to as protein homeostasis, or “proteostasis”.

### 2.1. Protein Quality Control at the ER: The Roles of Molecular Chaperones and the Proteostatic Network

As previously noted, CFTR is a relatively large monomeric protein. This is in stark contrast to most other ion channels, which are multimeric. The natively folded state of CFTR channels therefore consists entirely of intramolecular rather than intermolecular interactions. As a single CFTR polypeptide takes approximately 10 min to be fully translated [57], folding between domains primarily occurs co-translationally [58]. However, some of the most crucial stabilizing inter-domain interactions, such as those between NBD1 and NBD2, cannot be established until translation is complete [59,60]. As a result, CFTR lingers at the ER in a partially folded, energetically unfavorable state, during which it is especially vulnerable to targeting by components of the PQC machinery.

**Figure 2:**
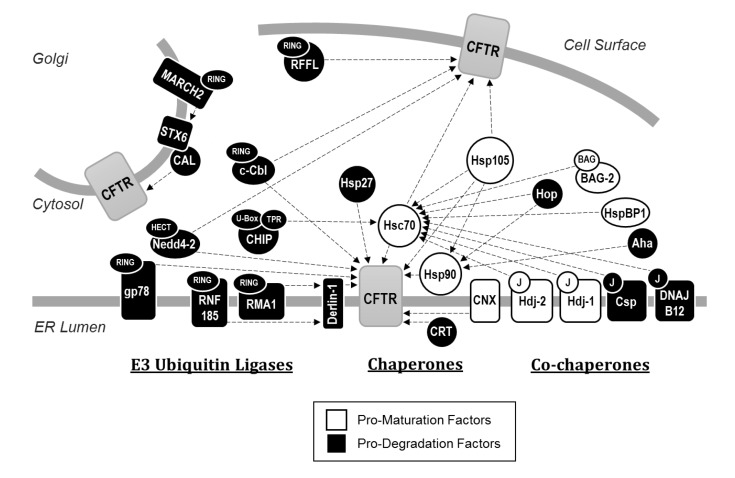
Network of CFTR PQC interactors. In broad terms, interaction partners sort into one of three groups: chaperones, co-chaperones, and E3 ubiquitin ligases. Each component within the network either enhances maturation of CFTR to the cell surface, or hinders maturation by selecting channels for degradation, although select factors share both pro-maturative and pro-degradative traits. While chaperones and co-chaperones vary widely in their effect, E3 ubiquitin ligases obligately facilitate degradation of CFTR through either the 26S proteasome or lysosome. Factors with an effect reported on either wild-type or F508del CFTR are depicted. Domains required for protein interactions and/or enzymatic activities are additionally depicted. Note that only select interactors are discussed at length.

CFTR PQC requires factors that drive the channel toward one of two diametrically opposed cellular fates (Figure 2). Some factors bind to CFTR to promote folding and maturation, whereas other factors recognize unfolded CFTR and instead direct these misfolded, potentially toxic channels for degradation. Though some factors bear both pro-maturative and pro-degradative activities during the biogenesis of proteins at the ER, typically one of these two activities has a greater net effect [61]. 

Molecular chaperones (also known as heat-shock proteins, or Hsps) are among the first components of the cellular PQC machinery to interact with CFTR, frequently acting co-translationally. These include Hsp90 and both the constitutively expressed and stress inducible isoforms of Hsp70, respectively referred to as Hsc70 and Hsp70 [62,63,64,65,66]. Hsc70, Hsp70, and Hsp90 bind CFTR and support folding through cycles of ATP hydrolysis. Specifically, their ablation in cell culture model systems causes nascent channels to become terminally misfolded and degraded. Although each of these chaperones nominally supports CFTR folding, Hsp70 and Hsp90 recruit numerous other PQC components, many of which antagonize channel folding and maturation. For example, the Hsp70/Hsp90 organizing protein (HOP) has an overall pro-degradative effect upon CFTR by drawing CFTR-bound Hsp90 into interactions with Hsc70 or Hsp70, and in turn with CHIP, an E3 ubiquitin ligase which directs the channel for degradation (see below). These events titrate CFTR off a pro-folding pathway into a pro-degradative pathway. As anticipated, inhibition of HOP favors CFTR maturation [67,68]. In contrast, HspBP1, a nucleotide exchange factor for Hsc70 and Hsp70, directly inhibits Hsc70 or Hsp70-bound CHIP and helps keep nascent CFTR on the pro-folding pathway [69,70].

Other Hsc70 co-chaperones, chiefly Hsp40 co-chaperones (also known as J-proteins), vary widely in their effects during the early maturation of CFTR. The ER-integral Hsp40 Hdj2 (DNAJA1) was the first member of the Hsp40 family recognized to bind CFTR and support productive folding [63]. While Hsc70 was initially observed to independently facilitate NBD1 folding in vitro [71], this chaperone was found to utilize both Hdj2 as well as a second Hsp40, Hdj1 (DNAJB1), to effectively rescue ER-retained wild-type, but not F508del CFTR from degradation [72]. However, while Hsp40s like Hdj2 or Hdj1 are required for efficient CFTR folding, these co-chaperones can also act as elements of the degradative PQC machinery. For example, in spite of its role in folding CFTR, Hdj2 can sharply increase the ubiquitin ligase activity of the Hsc70/CHIP complexes, as evidenced upon examination of CFTR sub-domains [73]. Another Hsp40, cysteine string protein (Csp, DNAJC5) facilitates degradation of CFTR due to its ability to independently recruit CHIP [74,75,76]. Similarly, DNAJB12 stimulates degradation of both immature forms of wild-type and F508del CFTR, but does so by recruiting another E3 ubiquitin ligase, RMA1, to Hsc70 [77,78]. The role of Hsp40s during degradation was also evidenced from studies in model systems. For example, the yeast ER-localized Hsp40 homologues Ydj1 and Hlj1 function redundantly to contribute to the degradation of ectopically expressed CFTR [79].

In contrast to the pro-degradative effects of Hsp70 and its broader co-chaperone network [80], Hsp90 is an important pro-folding chaperone, as perturbation of its function through either its deletion in the yeast model or its inhibition in human cell culture using small molecules prevents correct assembly of CFTR cytosolic domains and causes the nascent channels to be degraded [62,79]. In contrast, small heat shock proteins (sHsps) play a more complex role during wild-type and F508del CFTR biogenesis. These chaperones, which lack the ATPase domains of their larger counterparts but exhibit potent misfolded protein “holdase” activity [81], were initially implicated in the PQC of CFTR because deletion of the yeast sHsps, Hsp26 and Hsp42, greatly slowed CFTR degradation without altering the attachment of ubiquitin to the ER-retained channel [82] (see below). As expected, overexpression of the human sHsp αA-crystallin (HSPB4) in human cell culture accelerated degradation of F508del CFTR. Later studies indicated that a second human sHsp, Hsp27 (HSPB1), stimulated F508del CFTR degradation but did so by binding to incompletely folded NBD1 and recruiting Ubc9, an enzyme that catalyzes the attachment of small ubiquitin-like modifier (SUMO) [83,84]. In some cases, SUMO addition then leads to ubiquitination [83]. Consistent with these data, when the folding of NBD1 was improved, there was reduced SUMO addition, suggesting that sHsps primarily target incompletely folded conformations of CFTR.

In parallel, studies by Balch and colleagues used proteomic techniques to elucidate the broader CFTR “interactome”, revealing PQC elements that had not been previously revealed [85]. These included Aha1, a co-chaperone of Hsp90 that enhances the chaperone’s ATPase activity and displays an overall pro-degradative effect, perhaps because its action decouples Hsp90 from nascently folded CFTR and leaves the protein in a state where it is more vulnerable to co-translational degradation [86]. These studies also revealed the involvement of FKBP8, an ER localized peptidylprolyl isomerase that is specifically upregulated upon expression of F508del CFTR and recruited to chaperone complexes. FKBP8 mediates the *cis-trans* interconversion of proline residues necessary for CFTR to attain a trafficking-competent conformation [87]. Yet another CFTR-associated chaperone identified was Hsp105, which is a nucleotide exchange factor for Hsc70 [88]. Hsp105 stimulates the co-translational degradation of CFTR while also enhancing its post-translational maturation [89]. Together, it is clear that a spectrum of primary and secondary chaperone-associated partners is required to fold CFTR.

In contrast to wild-type CFTR, F508del displays a vastly shifted interactome, characterized by an increase in associated chaperones and co-chaperones, including many that target the channel for degradation [85]. Remarkably, early data indicated that prolonged hypothermia restores F508del CFTR processing and function [90] and does so by remodeling the interaction network to more closely resemble that of wild-type [91]. These data suggested that modifying the interactome, or “proteostatic network”, could achieve the same feat.

### 2.2. The Targeting of CFTR for Endoplasmic Reticulum Associated Degradation (ERAD)

Like other membrane proteins, CFTR enters the ER and becomes embedded into the ER membrane after entry through the Sec61 protein conducting channel [92,93,94]. More specifically, when polytopic integral membrane proteins such as CFTR enter Sec61, stretches of hydrophobic sequences corresponding to transmembrane spans fail to thread completely into the ER lumen but are instead released through a lateral gate in Sec61 and integrate into the membrane, leaving adjacent hydrophilic domains exposed to either the cytosol or ER lumen [95]. In contrast, as soluble luminal domains of CFTR enter the ER, the protein is N-glycosylated at two asparagine residues (N894 and N900) after synthesis of TMD2 [96]. As a result of this modification, CFTR may become subject to PQC decision-making by the lectin-like chaperones calnexin (CNX) and calreticulin (CRT) [97,98]. Though each of these chaperones is involved in the binding and retention of incompletely folded N-linked glycoproteins in the ER, their precise effects upon CFTR differ. CNX, an integral membrane chaperone in the ER, appears to facilitate the correct folding of the TMDs [99]. While its prolonged binding impedes maturation of channels from the ER, CNX may also shield incompletely folded forms of CFTR from recognition by pro-degradative PQC elements [100,101]. Even so, inhibition of CNX heavily favors the trafficking of wild-type CFTR from the ER but provides no benefit to the maturation of F508del CFTR. These results suggest that severely misfolded variants, like F508del, are generally targeted for degradation prior to the recognition of glycosylated asparagine residues [102]. In contrast, CRT, a soluble chaperone in the ER lumen, may facilitate CFTR turnover, perhaps because it extends ER dwell time without actively contributing to domain folding [98].

Although the degree of wild-type or F508del protein that folds might be both cell type and species specific [103,104,105], it is clear that the complex folding itinerary in the ER results in a significant amount of degradation of even the wild-type form of CFTR. In fact, even with the aid of the many identified pro-folding chaperones, perhaps only one-third of wild-type and virtually no F508del CFTR manages to attain a conformation that can be successfully trafficked from the ER; the remaining protein is instead degraded by the ubiquitin-proteasome system [57,96,106]. Misfolded channels selected for ubiquitin-proteasome-dependent degradation are handled by the ER Associated Degradation (ERAD) pathway. After recognition by pro-degradative chaperones, such as those outlined above, misfolded CFTR in the ER becomes covalently attached to chains of a 76-amino acid polypeptide, known as ubiquitin, which signals CFTR extraction from the ER membrane. The extraction, or “retrotranslocation”, of CFTR from the ER membrane requires an AAA-ATPase, known as p97, which then hands the protein off to cytosolic or ER-associated 26S proteasomes [106,107]. It is unclear whether CFTR is directly retrotranslocated from the ER membrane or whether it first enters a putative retrotranslocation channel, although interactions with some of these channels have been observed [49,92,94,108,109,110,111]. Ultimately, CFTR is hydrolyzed by the proteasome, which contains three unique proteolytic activities. Interestingly, only one of these activities, the chymotrypsin-like activity, is primarily required for CFTR turnover [112].

Several enzymes are required for the polyubiquitination of ERAD substrates, such as CFTR [113,114]. First an E1 ubiquitin activating enzyme must covalently bind ubiquitin in an ATP-dependent manner before transferring the activated ubiquitin to one of dozens of distinct E2 ubiquitin conjugating enzymes. These E2 enzymes, in turn, act in conjunction with specific E3 ubiquitin ligases to attach the ubiquitin moiety to lysine sidechains on suitable substrate proteins, or onto lysine sidechains of ubiquitin itself to extend the growing polyubiquitin chain. In the case of these isopeptide polyubiquitin linkages, the use of distinct internal lysine residues determines chain topology and cellular function. Canonically, polyubiquitin chains linked through K48 or K11 designate substrate proteins for degradation by the proteasome, whereas K63 linkages typically act as an endocytic signal [115]. While it is unclear exactly which linkage type(s) mark CFTR for ERAD, or whether branched or mixed linkage chains are utilized (which appear to be quite common [116]), ubiquitination of certain lysine residues within the channel (specifically K14, K68, and K1218) preferentially selects CFTR for degradation [117].

To date, >600 putative E3 ubiquitin ligases have been identified in the human genome [118]. While some of these enzymes are substrate-specific, most are thought to target multiple substrates, especially during ERAD [116]. Therefore, each protein can be ubiquitinated by multiple E3s. Indeed, to date four ubiquitin ligases, RMA1, gp78, CHIP, and RNF185, have been conclusively shown to mediate the ERAD of CFTR.

A significant body of data indicates that RMA1, gp78, CHIP, and RNF185 act at different steps during CFTR biogenesis. Early studies by Kopito and colleagues revealed that CFTR is translated within 30 min but ubiquitination of both wild-type and F508del CFTR begins to appear after only 20 min, indicating that ubiquitination occurs concurrently with translation, at least in an in vitro reticulocyte lysate [119]. Consistent with these data, the earliest acting ubiquitin ligases on CFTR are a pair of ER integral membrane proteins, RMA1 (also known as RNF5) and its highly conserved homologue, RNF185, which target immature or misfolded forms of CFTR immediately after NBD1 translation [49,120]. Neither enzyme is capable of binding CFTR directly, but do so through Derlin, an integral membrane adaptor protein that—as noted above—has been suggested to act as a retrotranslocation channel and also assembles multiple components of the ERAD machinery [121,122,123,124,125]. While RMA1 and RNF185 readily target both wild-type and F508del CFTR intermediates for degradation [49,120], RMA1 ablation more prominently improves F508del CFTR maturation and can partially alleviate a CF phenotype in mice, even without additional therapeutic interventions [126]. Consistent with data that E3 ubiquitin ligases work with one another [116], a third ER-integral E3 ubiquitin ligase, gp78, augments the activity of RMA1 and RNF185 [127] by predominantly elongating polyubiquitin chains initiated by either enzyme and contributes to the ERAD of F508del CFTR. In contrast to these early acting E3 ubiquitin ligases, the cytosolic ubiquitin ligase CHIP is most active on fully translated CFTR [49]. Like RMA1 and RNF185, CHIP is unable to bind misfolded CFTR channels directly, but in this case it does so by binding Hsc70 (or Hsp70) through an EEVD motif present at the carboxyl terminus of the chaperone. Thus, CHIP co-opts the activity of otherwise pro-folding chaperones to instead trigger ERAD [128,129]. Interestingly, although Hsp90 shares a similar carboxyl-terminal motif, CHIP only stimulates the ERAD of CFTR through its interaction with Hsc70 or Hsp70 [129].

### 2.3. Trafficking of CFTR from the ER

In order to evade ERAD and traffic from the ER, CFTR must pass several of the PQC checkpoints noted above. First, the channels must be sufficiently folded to be released by CNX and CRT. Substrate binding and release of CNX/CRT-substrate complexes depends on the configuration of the glycan, a branched polysaccharide that is gradually trimmed as secretory proteins dwell in the ER [130]. CNX and CRT bind glycans with a single terminal glucose but release glycoprotein substrates after this glucose is cleaved by α-glucosidase II. In turn, nascent proteins that have failed to acquire their native conformations can be re-glucosylated by an enzyme that monitors protein conformation. Second, CFTR folding conceals four partially redundant arginine-framed tripeptide (AFT) sequences located within the N-terminus, NBD1, and R domain of CFTR, which facilitate the retention of CFTR in the ER if exposed to the cytosol [131]. Although mutagenizing these motifs favors F508del CFTR trafficking and function at the cell surface in cell culture, their removal has no discernable impact upon the processing of wild-type CFTR. This result suggests that AFTs alone are not responsible for the retention and degradation of the majority of these channels. Finally, a di-acidic motif (YKDAD) on the surface of NBD1 must be engaged at ER exit sites by two secretory trafficking components, Sec23/Sec24 [132,133], which function as inner coat components of COPII vesicles that mediate ER-to-Golgi transport of secreted cargo proteins [134]. Thus, after recognition, properly folded forms of CFTR are sorted into COPII-coated vesicles, which bud from the ER. In the Golgi, CFTR is further modified by *O*-linked glycosylation *en route* to the cell surface [96,135].

Studies from the analysis of numerous intragenic suppressor mutations in the gene encoding F508del CFTR led to the concept that more than one mechanism is needed to “fix” the folding defect and allow for ER exit. While misfolding within NBD1 is the primary folding defect caused by the absence of F508 [50], this mutation also disrupts the interaction between NBD1 and TMD2 at highly conserved residues in intracellular loop 4 (ICL4) [59,136]. Notably, distinct second-site suppressors could restore favorable folding within NBD1 or interdomain assembly between NBD1 and TMD2. While the presence of either suppressor alone proved insufficient to restore maximal F508del CFTR assembly and function, when these distinct suppressor mutations were combined they acted synergistically to produce CFTR channels that folded, exited the ER, and functioned at the cell surface. This finding signaled that a single compound designed to facilitate the folding of a specific domain in F508del CFTR or other misfolded variants would be unable to fully mend the aberrant channel. The developments leading to subsequent combinatorial therapies are discussed further in Section 3.

In contrast to the forward trafficking pathway, CFTR can also undergo anterograde trafficking through an unconventional pathway when cells are exposed to ER stress, which disrupts ER-to-Golgi transport. During stress, activation of IRE1, which initiates the unfolded protein response (UPR), increases both the number of ER exit sites and expression of Sec16A, a secretory protein concentrated at these sites that facilitates scaffolding of COPII-coated vesicles [137,138]. Concurrently, the Golgi-resident protein GRASP55 is phosphorylated, which causes it to dissociate from homodimers into monomers that traffic to the ER where they interact with Sec16A [138,139]. Through a mechanism that remains incompletely understood, GRASP55/Sec16A drives the export of CFTR directly to the cell surface, apparently bypassing the Golgi since CFTR trafficked under these conditions lacks Golgi-associated glycosylation. Fascinatingly, the unconventionally trafficked F508del CFTR protein is sufficiently functional, as transgenic mice expressing GRASP55 lack phenotypes associated with the F508del allele, suggesting that modulation of this pathway could provide therapeutic benefit [140]. Earlier hints that CFTR bypasses the Golgi apparatus also emerged from work in which the protein was shown to leave the ER even in spite of overexpressed dominant negative versions of required COPII trafficking regulators, yet remained dependent upon fusion with endosomes [132]. Which proteins coat CFTR-containing vesicles emerging from ER exit sites and how these vesicles interact with cytoskeletal components in this unconventional pathway remain to be elucidated.

### 2.4. Post-ER Quality Control: Targeting of Plasma Membrane and Endosomal CFTR for Lysosomal Degradation

After its delivery to the cell surface, CFTR acts as an anion channel with a moderate open probability after phosphorylation (wild-type P_0_ ≈ 0.4) [54,55]. Even after residing in its ultimate site of action, the channel remains acutely sensitive to PQC components. To this end, a tyrosine-based endocytic motif within the C-terminus of CFTR (YXXΦ) [141,142,143] signals the sorting of CFTR into clathrin-coated pits through direct binding with the adaptor protein AP-2 [144,145,146]. After subsequent budding from pits into clathrin-coated vesicles, binding between Dab2 and AP-2 links vesicles to myosin IV for transport from the plasma membrane to post-endocytic compartments [147,148,149,150]. CFTR residing in endosomes is then either transported back to the cell surface via recycling endosomes, or is selected by PQC components for trafficking to the lysosome through late endosomes and multivescicular bodies (MVBs). Similar to ERAD, selection for lysosomal trafficking and degradation is signaled by CFTR polyubiquitination. Interestingly, if F508del CFTR folding is restored (e.g., by low temperature correction; see Section 3.1), which facilitates accumulation of the protein at the cell surface, PQC components recognize the channel more readily than wild-type CFTR [151,152]. As a result, F508del CFTR is endocytosed faster than the wild-type channel. Interestingly, there is some evidence that folding correctors also stabilize F508del CFTR at the cell surface [153]. Regardless, because the mutant channel also exhibits a substantially lower P_0_ than the wild-type protein at the cell surface [54,55], for clinical benefit small molecule correction of F508del CFTR folding must be accompanied by drugs that also improve channel gating (see below).

Two E3 ubiquitin ligases have been implicated in the ubiquitination of CFTR at the cell periphery. Just as in ERAD, cytosolic CHIP/Hsc70 complexes recognize misfolded F508del CFTR in endosomes and recruit components of the ubiquitination machinery to mark these channels for lysosomal degradation [152]. Interestingly, CHIP activity on CFTR in the late secretory pathway is abrogated by the loss of endocytic factors, such as Dab2, and occurs ~15 min after internalization [150]. This observation suggests that CHIP acts upon CFTR in endosomes, but not at the cell surface. A second cytosolic E3 ubiquitin ligase, RFFL, is also involved in the turnover of CFTR residing at the cell surface. Unlike CHIP, RFFL is palmitoylated and interacts directly with CFTR, binding through disordered regions independent of molecular chaperones [154]. Ablation of RFFL in cell culture has no effect on the turnover of wild-type CFTR at the cell membrane, indicating that the ligase specifically targets mutant CFTR variants at the cell surface.

The C-terminus of CFTR also binds to numerous PDZ proteins, such as NHERF1 [155], which stabilize and limit the membrane mobility of both wild-type and rescued F508del CFTR at the cell surface [156,157]. One notable exception is the CFTR-associated ligand (CAL), which binds peripheral CFTR and targets it for lysosomal degradation by recruiting a SNARE protein, syntaxin 6 (STX6) [158,159,160]. Additionally, CAL has been reported to localize to the ER where it contributes to the ERAD of F508del CFTR [161]. In principle, effective modulators that inhibit binding of CAL to CFTR are anticipated to have therapeutic benefits [162], and to this end both peptide and small molecular interventions that block PDZ domain binding have been explored [162,163].

## 3. Pharmacological Interventions in Cystic Fibrosis

As mentioned in Section 1, CF therapies initially focused on alleviating disease symptoms rather than rectifying its core molecular defect: the dysfunction of CFTR channels. However, the discovery of CFTR as the genetic determinant for disease, in conjunction with subsequent elucidation of the proteostatic pathways that regulate the folding, degradation, trafficking, and function of the channel, have led to the development of therapeutics that directly modulate the biogenesis and activity of otherwise defective CFTR variants.

### 3.1. Correction of CFTR Misfolding Promotes Forward Trafficking

Although class II CFTR variants are retained in the ER and targeted for ERAD (see above), simply shifting cells to a lower temperature corrects the misfolding defect for many variants, including F508del, and enables the release of maturely glycosylated, partially functional channels to the cell surface [90,164]. Though of limited therapeutic benefit, this observation was significant because it implied that if mutant CFTR could be rescued by hypothermia, it might also be rescued by treatment with a compound or set of compounds that successfully recapitulates the effect of low temperature. Welsh and colleagues were the first to provide proof-of-concept for this hypothesis, demonstrating that chemical chaperones, such as glycerol and trimethylamine N-oxide (TMAO), which favor protein folding in vitro, restored F508del CFTR processing and activity when added to cultured cells at 37 °C [165,166,167]. Over time, other osmolytes and chemical chaperones were identified that conferred similar protective effects [168].

Subsequent studies sought compounds that also thermodynamically stabilized F508del CFTR but could be tolerated *in vivo*. One such chemical chaperone, 4-phenylbuterate (4PBA), emerged as an early candidate as it had already received regulatory approval for the treatment of urea cycle disorders. Indeed, high micromolar treatment with 4PBA restored functional maturation of F508del CFTR in cell culture [169]. Moreover, 4PBA appeared to interfere with the selection of F508del CFTR for degradation by downregulating Hsc70 [170]. While early trials of 4PBA in CF patients showed that it was generally well tolerated and quantifiably stimulated channel activity, it conferred only a slight benefit on respiratory function [171,172]. Clearly, more specific modulators would be needed to successfully resolve the misfolding defect caused by F508del and similar malfunctioning alleles.

The advent of massive libraries of drug-like compounds and the development of a fluorescence-based cell culture assay for CFTR activity that was suitable for high throughput screening (HTS) made it possible to query tens of thousands of compounds, leading to the identification of CFTR modulators that exhibited effects at low micromolar concentrations [173]. These modulators sorted into one of two conceptual classes: “corrector” compounds, which improve CFTR function by augmenting folding and trafficking of the channel, and “potentiator” compounds, which improve the frequency at which the channel gates to conduct anions. Subsequent screening using a modified assay, in which CFTR was rescued by low temperature prior to screening, identified several dozen CFTR potentiators that were structurally unrelated to correctors and previously identified potentiators [174].

Ongoing HTS efforts led to the discovery of ever more potent CFTR correctors. One compound was Corr-4a, the first corrector to restore as much F508del CFTR function at 37 °C as that restored by incubation of untreated cells at 27 °C [175]. Studies by Vertex Pharmaceuticals then identified VRT-422 and VRT-325, which restored F508del CFTR-mediated chloride current to ~10% of wild-type CFTR current in human bronchial epithelial (HBE) cells, a level associated with a mild CF phenotype [176]. Further medicinal chemistry efforts by the company bore VX-809, which rescued ~30% of F508del CFTR from ERAD and restored ~14% of wild-type chloride secretion; the compound also lacked the off-target effects associated with other compounds [177]. Additional studies indicated that VX-809 interacted directly with nascent channels, most likely by binding at the interface between NBD1 and TMD1 to stabilize early folding intermediates during translation [178,179]. Even so, VX-809 does not correct every misfolding defect associated with F508del as it displays additive effects with Corr-4a, VRT-325, low temperature, and with the introduction of several folding suppressor mutations into F508del CFTR [180].

### 3.2. Potentiation of Channel Conductance

Compounds that potentiate the anion transducing activity of cell surface CFTR channels were well known prior to the identification of folding correctors. One such early potentiator was genistein, a plant product that closely resembles estradiol. When combined with forskolin, a cAMP agonist, enhanced CFTR conductance was observed in cell culture [181]. Although a portion of this effect was proposed to stem from the action of genistein as a tyrosine kinase inhibitor, possibly preventing CFTR dephosphorylation [182], later electrophysiological studies revealed that even in excised cell membrane patches, genistein enhanced conductance of both wild-type and F508del CFTR [56].

Potentiators initially identified through HTS strategies had no effect on the forward trafficking of F508del CFTR but improved gating of low temperature-rescued F508del channels as effectively as genistein, yet with ~10-fold higher affinity [174]. A subsequent HTS conducted by Vertex Pharmaceuticals led to the identification of VRT-532, a potentiator that also improved F508del CFTR conductance but additively improved conductance when combined with correctors, such as VRT-422 or VRT-325. These data supported the notion that misfolding and inadequate gating arise from distinctly different defects caused by the F508del mutation, and that fixing each defect would require dedicated modulators. Additional examination of VRT-532 indicated that the compound directly bound F508del CFTR channels, as predicted [183].

Further efforts to refine the pharmacology of VRT-532 and other hits from earlier potentiator screens resulted in the isolation of VX-770 [54]. Approximately 70 times more potent than genistein, VX-770 acted as a potentiator for CFTR variants regardless of folding propensity, enhancing the open probability of wild-type, F508del, and G551D CFTR channels in excised patches. Additionally, low micromolar quantities of VX-770 increased the height of airway surface liquid in F508del/G551D human bronchial epithelia (HBE) cultures from one-quarter to one-half of that in control HBE. This result signified that the potentiator might meaningfully counteract dehydration of the mucous layer in CF epithelia harboring at least one trafficking-competent CFTR variant. Recent breakthroughs in determining the molecular structure of CFTR channels [28,29,32] have lent support to the notion that VX-770 mediates channel activity via hydrogen bonding to the transmembrane domains of CFTR, as does an unrelated potentiator developed by Galapagos Pharmaceuticals. Collectively, it appears that binding to transmembrane domains is a general mechanism of action amongst CFTR potentiators [184,185].

### 3.3. Compounds Currently in Clinical Use

VX-809 and VX-770 (subsequently re-identified as lumacaftor and ivacaftor, respectively) entered clinical trials soon after their isolation. Trials with VX-770 concluded that the potentiator provided substantial benefit to respiratory function and overall quality of life among CF patients 12 years and older with at least one G551D CFTR allele, a classical class III variant [186]. VX-770 received FDA approval in 2012 and was then marketed as Kalydeco^™^, the first small molecule modulator designed to directly address an underlying molecular defect in CF. Studies published soon after indicated that VX-770 similarly improved the in vitro conductance of a broad range of class III and class IV CFTR variants, opening the door for VX-770 to be tested in patients bearing these alleles as well [51]. VX-770 furthermore improved conductance of a number of class II variants, beyond F508del [187]. However, in spite of its functional benefit in various cell culture models, treatment with VX-770 alone proved ineffective in patients homozygous for F508del [188], confirming that a corrector/potentiator combination therapy would be needed to address the unique challenges presented by class II mutations.

Upon subsequent clinical testing, a VX-809/VX-770 combination, marketed as Orkambi^™^, received FDA approval in 2015, making CFTR modulator therapy available to a broader cross-section of CF patients. However, the benefit that this drug cocktail provided patients with F508del mutations paled in contrast to that which VX-770 provided to G551D patients. While Kalydeco^™^ appeared to deliver a “functional cure” for patients encoding even a single gating mutation, Orkambi^™^ provided F508del homozygous patients only a 4% enhancement on average of one second forced expiratory volume (FEV_1_) [189]. Even so, the number of pulmonary exacerbations in those taking Orkambi^™^ fell from ~1.1 to ~0.8 over a 48 week time frame. This relatively modest benefit, combined with the fact that VX-809 and VX-770 exhibited antagonistic effects [190,191], highlighted the need for additional therapies to successfully modulate CFTR in most patients.

Continued adjustment of corrector therapies led to the development of two next generation CFTR correctors: VX-661 (tezacaftor, a VX-809 mimic with an improved pharmacological profile) and VX-445 (elexacaftor), the latter of which corrects F508del CFTR via a mechanism of action that is distinct from VX-809 and VX-661. Although VX-770 continued to diminish the quantity of mature CFTR rescued by treatment with these correctors and needs to be taken twice daily, the VX-445/VX-661/VX-770 corrector/potentiator combination considerably improved patients’ wellbeing, especially when compared to Orkambi^™^ [192]. In an early clinical trial of patients with either one or two copies of F508del CFTR, CF patients treated with this triple drug combination displayed a 10% improvement in FEV_1_ on average, along with a sharp, concomitant drop in sweat chloride and a significantly reduced frequency of pulmonary exacerbations. Subsequent phase III trials examining the effect of this drug combination on both F508del homozygous and heterozygous patients confirmed the substantial improvements seen in this cohort of CF patients [193,194]. Ultimately, the VX-445/VX-661/VX-770 combination received FDA approval for both patient groups in late 2019 and is now marketed as Trikafta^™^, replacing Orkambi^™^ as the gold standard for treatment of severely misfolded CFTR variants. Perhaps most importantly, Trikafta^™^ and Kalydeco^™^ provide benefits for >90% of the CF patient community.

## 4. The Path Forward in Cystic Fibrosis

Looking forward, there are several significant considerations for the future of CFTR modulator therapies. First, it appears increasingly important that patients with variants known to respond well to current therapies have access to these treatments from as young an age as possible. Modulation will clearly provide the greatest benefit if started before lung tissue becomes permanently damaged. To this end, therapies initially approved for patients 12 years of age or older have since been tested in younger children. Typically, these patients show similar responses to the drugs as when older patients are treated [195,196].

**Figure 3:**
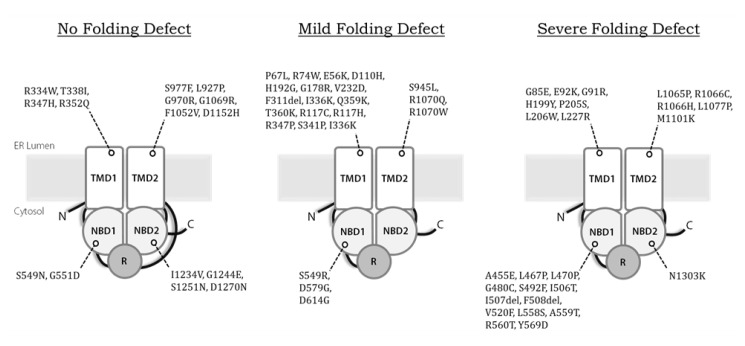
Disease-causing variants of CFTR by channel folding severity. While severely misfolding variants such as F508del are classified as class II mutations with additional gating and/or conductance defects and are associated with severe CF phenotypes, function of many of these channels could be restored with currently available triple drug combination therapies. Illustration is not intended to depict structures of individual variants.

Second, while we estimate that disease-causing alleles currently approved for treatment with Kalydeco^™^ or Trikafta^™^ together encompass 70–80% of CF patients in the United States, Canada, and Europe (https://cftr2.org), patients who encode other variants continue to be left with therapies that alleviate only the symptoms of CF, but not the cause. Therefore it remains vital to continue to “theratype” rare alleles according to their pharmaceutical responses [197]. This is especially important for variants that may all appear to exhibit similar defects but may differ radically in the way they interact with correctors and potentiators. For example, F508del, E92K, and G85E CFTR are all class II variants that exhibit severe misfolding defects (Figure 3), yet only F508del and E92K are approved for treatment. In contrast, G85E is completely intractable to correction, whether by low temperature or by treatment with small molecules [164]. The nature of this phenomenon remains mysterious. Of particular concern are the CFTR truncation mutations or splice site variants, which can produce catastrophically altered/misfolded channels, or at best might be partially rescued, as observed with the W1282X allele [198]. Ongoing efforts have been dedicated to the identification and clinical evaluation of compounds that promote translational read-through or genetic strategies that silent aberrantly spliced products [199].

Third, the long term efficacy of pharmacological correctors and potentiators remains unknown. While current therapies remain relatively new, data for Kalydeco^™^ have already emerged indicating that VX-770 slows the rate of respiratory decline, but fails to prevent it [200]. Whether the same is true for Trikafta^™^ remains to be seen. In addition, the current annual price for Trikafta^™^ is > $300,000. One hopes that the development and approval of other drug combinations that provide patient benefits will lead to a reduction in the cost of CF therapeutics, improving the availability of these therapies worldwide. Moreover, because VX-445 (elexacaftor) has been tested in patients for a significantly shorter time compared to the other drugs in the cocktail, its long-term effects and any potential negative drug-drug interactions are unknown. Finally, it is unfortunate that not every symptom associated with CF will necessarily be cured by modulator combinations. Ultimately, techniques that edit or replace dysfunctional CFTR genes could provide a permanent cure for all CF patients regardless of allelic combination, bypassing the need for pharmacological modulators altogether.

Until gene editing becomes common, however, it remains imperative that a continued analysis of the proteostatic pathways to which CFTR and disease-causing alleles are subject is continued. In support of this view, studies on the rules governing the identification and disposal of CFTR alleles have yielded surprises. In addition to the non-classical secretion mechanisms noted in the text, some disease-causing alleles appear to be disposed of by the autophagy pathway [201,202], and defects in this alternate degradation pathway have been linked to the expression of CF-associated alleles [203]. In addition, modulation of the proteostatic pathways that impact CFTR folding, stability, degradation, and trafficking might yield new therapeutic opportunities. Notably, if ERAD of CFTR is slowed through the use of a small molecular inhibitor of the ubiquitin pathway, synergistic effects on F508del maturation and activity are evident when lung epithelial cells are treated with a corrector [204]. A better understanding of the proteostatic pathways in CF are warranted because small molecule modulators of these pathways are actively being identified and characterized [205,206].
